# Neural Responses to Smoking Stimuli Are Influenced by Smokers' Attitudes towards Their Own Smoking Behaviour

**DOI:** 10.1371/journal.pone.0046782

**Published:** 2012-11-14

**Authors:** Bastian Stippekohl, Markus H. Winkler, Bertram Walter, Sabine Kagerer, Ronald F. Mucha, Paul Pauli, Dieter Vaitl, Rudolf Stark

**Affiliations:** 1 Bender Institute of Neuroimaging, Justus-Liebig-University of Giessen, Giessen, Germany; 2 Department of Psychology, Julius-Maximilians-University of Würzburg, Würzburg, Germany; Centre for Addiction and Mental Health, Canada

## Abstract

An important feature of addiction is the high drug craving that may promote the continuation of consumption. Environmental stimuli classically conditioned to drug-intake have a strong motivational power for addicts and can elicit craving. However, addicts differ in the attitudes towards their own consumption behavior: some are content with drug taking (consonant users) whereas others are discontent (dissonant users). Such differences may be important for clinical practice because the experience of dissonance might enhance the likelihood to consider treatment. This fMRI study investigated in smokers whether these different attitudes influence subjective and neural responses to smoking stimuli. Based on self-characterization, smokers were divided into consonant and dissonant smokers. These two groups were presented smoking stimuli and neutral stimuli. Former studies have suggested differences in the impact of smoking stimuli depending on the temporal stage of the smoking ritual they are associated with. Therefore, we used stimuli associated with the beginning (BEGIN-smoking-stimuli) and stimuli associated with the terminal stage (END-smoking-stimuli) of the smoking ritual as distinct stimulus categories. Stimulus ratings did not differ between both groups. Brain data showed that BEGIN-smoking-stimuli led to enhanced mesolimbic responses (amygdala, hippocampus, insula) in dissonant compared to consonant smokers. In response to END-smoking-stimuli, dissonant smokers showed reduced mesocortical responses (orbitofrontal cortex, subcallosal cortex) compared to consonant smokers. These results suggest that smoking stimuli with a high incentive value (BEGIN-smoking-stimuli) are more appetitive for dissonant than consonant smokers at least on the neural level. To the contrary, smoking stimuli with low incentive value (END-smoking-stimuli) seem to be less appetitive for dissonant smokers than consonant smokers. These differences might be one reason why dissonant smokers experience difficulties in translating their attitudes into an actual behavior change.

## Introduction

Tobacco addiction is a chronically relapsing disorder characterized by withdrawal symptoms when abstinent, strong compulsions for drug-use (craving), and loss of control over intake [1]. As a result, addicts show a remarkable persistence of drug-use behavior despite its apparent negative consequences [2], [3]. However, after a while, they often experience a growing dissociation between the desire to consume a drug and the reflective evaluation of their behavior. As a result, addicts often want to quit drug consumption because of rational reasons but are unable to do so because of the overwhelming desire to consume and a severely reduced ability to control this desire [4], [5]. This dissociation has been hypothesized to result at least partly from a maladaptive interaction between two systems that are important for guiding behavior: an ‘impulsive’ system that is driven by signals of immediate reward and a ‘reflective’ control system which is sensitive to prospective positive or negative consequences [5], [6]-[9]. However, addicts differ considerably in their tendency to engage in reflective processing of the negative consequences of drug-taking and, as a result, in their attitudes towards their own consumption behavior.

Concerning nicotine addiction, McKennel & Thomas (1967, cited in [10]; see also [11]) introduced the concept of consonant and dissonant smokers to describe such differences in reflective processing and attitude. Consonant smokers are content with their smoking behavior, experience more advantages than disadvantages, and do not want to quit, even if this could be done easily. Dissonant smokers are discontent with their smoking behavior, experience more disadvantages than advantages, and want to quit. The distinction between these groups is important for clinical considerations because the development of dissonance is an important first step for changing addictive behavior [11], [12]–[16].

As described above, craving is a major hallmark of addiction. Importantly, it can be elicited by external or internal stimuli that are classically conditioned to drug intake [4], [17], [18]. These ‘drug-cues’ have been proposed to signal the immediate availability of drug reward, thereby evoking activity in the impulsive system, which is not sufficiently counteracted by the reflective system (e.g. [4], [5], [8], [9], [17], [18]). Therefore, they are believed to possess a strong motivational impact on addicts promoting continued consumption and relapses [18]–[20].

Human research has provided considerable evidence that drug-cues can elicit craving (for an overview see [21]), appetitive psychophysiological responses (e.g. [22]–[25]), and mesocorticolimbic brain activity underlying incentive affective processing (for overviews see [26]–[31]). Concerning nicotine addiction, however, recent studies with smokers have shown that this reactivity might depend on the temporal position of the stimuli in the consumption ritual [24], [32]–[35]. Stimuli associated with the *beginning* of the smoking ritual (BEGIN-smoking-stimuli) elicit high cue-reactivity as described above. In contrast, stimuli associated with the *terminal stage* of the smoking ritual (END-smoking-stimuli) seem to evoke only modest cue-reactivity. More precisely, END-smoking-stimuli fail to evoke the high craving response seen for BEGIN-smoking-stimuli [24], [32]. Further, whereas BEGIN-smoking-stimuli attenuate the startle response similar to positively valenced pictures, such an effect was not found for END-smoking-stimuli [22], [24], [33]. Moreover, END-smoking-stimuli fail to distract smokers from current activities (attentional bias), which is believed to be an important feature of drug-cues and can be observed for BEGIN-smoking-stimuli [35]. These differences could also apply to other addictions, because similar results were found for alcoholics and alcohol stimuli [36], [37].

Importantly, one should not prematurely assume END-smoking-stimuli to be simply weak cues, because Mucha and colleagues [24] demonstrated that END-smoking-stimuli seem to reduce the effects of BEGIN-smoking-stimuli when both are presented together. Further, Stippekohl et al. [34] found END-smoking-stimuli to elicit a neural response pattern that was composed of activations as well as deactivations. Deactivations occurred in the ventral striatum and the anterior cingulate cortex, which are believed to be involved in processes like cue detection, appetitive processing, craving, and the loss of control that characterizes addiction (e.g. [4], [18], [26], [27], [47]). Thus, responses elicited by END-smoking-stimuli might represent a unique reactivity, which may even oppose the responses triggered by BEGIN-smoking-stimuli. These findings may be surprising, because when terminal stimuli naturally occur, the blood nicotine level reaches its peak (see also [24], [38]–[40]). Thus, one could also assume that END-smoking-stimuli may be associated with higher levels of reward or pleasure. However, results of Mucha et al. [24] suggest that the differences between BEGIN- and END-smoking-stimuli might be due to differences in the signalled drug availability (high for BEGIN-smoking-stimuli, low for END-smoking-stimuli) and previous research has demonstrated that drug availability is an important modulator of subjects' responses to drug cues (stronger responses when drug availability is high [41]–[46]).

Despite a huge amount of research concerning both, attitudes towards smoking behavior and reactivity towards smoking stimuli, studies investigating the influence of the different attitudes on the responses to smoking stimuli are rare. Using the startle response as a physiological measure of affective state, current studies suggest that the attitude towards the ones own smoking behavior can have an influence on cue reactivity. While smoking cues elicit a positive affect in subjects not willing to quit (leading to an attenuation of the startle response), this effect seems to be reduced in subjects with a high motivation to quit [23], [48]. Additionally, smokers willing to quit showed higher heart rate responses and a stronger feeling of guilt in response to smoking cues [49]. However, data regarding subjective responses to cues are ambiguous. McDermut and Haaga [49] as well as Dempsey and colleagues [23] found no evidence of altered craving, valence, or arousal responses. Munoz et al. [48] to the contrary, found higher valence, lower arousal, and a trend for lower craving ratings in smokers with low motivation to quit compared to smokers with high motivation to quit.

Considering neural responses to smoking stimuli, the effects of different attitudes towards smoking have, to our knowledge, not yet been investigated. To account for this lack, we performed the present analysis of an fMRI data set that was collected in the course of a larger study to obtain information on how consonant and dissonant smokers differ in the processing of smoking related stimuli (BEGIN-smoking-stimuli as well as END-smoking-stimuli) on a subjective and neural level (see methods for details). We hypothesized that consonant smokers are more responsive to the impact of smoking stimuli than dissonant smokers, because the negative attitude towards one's own smoking behavior in dissonant smokers supposedly alters the responses to these stimuli. Specifically, we hypothesized that BEGIN-smoking-stimuli, which signal the immediate availability of a drug reward, trigger stronger subjective and neural responses in consonant smokers than in dissonant smokers. For END-smoking-stimuli, which signal only a low drug availability, we expected reverse results. END-smoking-stimuli signal the unavailability of a drug reward and should thus lead to avoidance behavior and further search for stimuli signaling the availability of a drug reward. These effects should be stronger for consonant smokers because they should be more motivated for smoking than dissonant smokers. Further, because END-smoking-stimuli have been shown to lead to reduced appetitive responses or to an inhibition of these responses, we expected this effect (reduction of responses) to be stronger in consonant smokers. This, in turn, should lead to weaker responses in consonant compared to dissonant smokers in the statistical group comparison.

## Methods

The current analysis is part of a larger study investigating the effects of seven different stimulus categories on neural activity in smokers and non-smokers.

### Subjects

Sixteen consonant and sixteen dissonant smokers participated in the study. The two groups were defined on the basis of criteria proposed by Eiser et al. [10]. First, a written definition of the terms “consonant smoker” and “dissonant smoker” (referring to Eiser, Sutton and Wober [10] and McKennel & Thomas, 1967 (cited in [10]) was provided.

Then, subjects were asked to characterize themselves on a 9-point Likert scale ranging form (1) “purely consonant” to (9) “purely dissonant”. Only subjects with values ranging from 1 to 3 (consonant smokers) or from 7 to 9 (dissonant smokers) were invited to participate in the study. The description of the two types of smoking attitudes read:

**Dear participants,**

the aim of this questionnaire is to determine your attitude towards your own smoking behavior and to you being a smoker. There are no right or wrong answers. It is merely about your personal opinion. In order to simplify the process, we will introduce two types of smoking attitudes, among which smokers vary: consonant smokers perceive their smoking behavior as very positive, dissonant smokers however perceive their smoking behavior as very negative. You will have to judge your own attitude on a 9-point scale indicating how much you tend to one of these types.

Smoking attitudes vary between the two types that will now be introduced in more detail:

1. **Consonant smokers:**
  - like smoking, are satisfied with their smoking habits and do not want to quit.
  - perceive more advantages than disadvantages in smoking, e.g., ‘Smoking is fun, relaxing, and helpful in stressful situations’.
  - would answer the question ‘Would you quit smoking, if you could do so easily?’ with ‘no’.
2. **Dissonant smokers:**
  - do not like smoking, are dissatisfied with their smoking behavior and would like to quit.
  - perceive more disadvantages than advantages in smoking, e.g., ‘I worry about my health, but do not manage to quit’.
  - would answer the question ‘Would you quit smoking, if you could do so easily?’ with ‘yes’.

All subjects were right handed. Most of them were students receiving either money (10 Euro/h) or course credits for their participation. No subject was taking regular medication, had a history of psychiatric or neurological illness, or reported other drug abuse. All subjects were fully informed about the experimental procedure and gave their written consent. Regarding the purposes of the study, they were told that neural and subjective responses to smoking and general emotional stimuli would be investigated. To avoid biases in our data, subjects were not informed about any potential influences of the attitude towards their own smoking behavior prior to the experiment. The study was performed in accordance with the ethical standards of the fifth revision of the Declaration of Helsinki and approved by the Ethical Committee of the German Psychological Association.

### Stimuli and task

Seven stimulus categories were presented: smoking stimuli and their respective control stimuli were taken from a picture set developed previously [34]. Smoking cues (BEGIN-smoking-stimuli) depicted the beginning of the smoking ritual and showed someone taking the first puff on a cigarette. Pictures showing the terminal stage of the smoking ritual (END-smoking-stimuli) depicted someone stubbing out a cigarette butt. Control pictures for BEGIN-smoking-stimuli showed someone putting a toothbrush into the mouth; control pictures for END-smoking-stimuli depicted someone putting a toothbrush back into a beaker. Smoking and control pictures were close-up views of the respective actions. Pictures of the teeth-brushing ritual were used as control condition, because they can be matched to the smoking process with respect to temporal stages, body parts, as well as number and color of objects shown (for example, ashtrays, lighters, toothbrushes, beakers). Teeth-brushing can be considered as neutral because it is an overlearned everyday routine. This assumption was shown to be true in former studies, which yielded neutral ratings for the teeth-brushing stimuli [34], [35]. Further emotional stimuli presented during the experiment but not analyzed here were aversive pictures (accidents, mutilations etc.), erotic pictures, and appropriate control stimuli showing humans in everyday activities. These stimuli were included to investigate whether smokers differ from non-smokers in the (neural) processing of general emotional stimuli. The stimuli were taken from the International Affective Picture System (IAPS [50]) and an own picture collection. This is the first analysis of the overall data set. The data regarding the smoking stimuli will be the focus of this paper. Results regarding the other emotional stimuli will be reported separately. All stimuli were presented in 800×600 pixel resolution. An LCD projector (EPSON EMP-7250) projected them onto a screen at the end of the scanner (visual field = 18°) where they were viewed through a mirror mounted on the head coil.

Stimuli were presented in 28 randomly arranged blocks, with 4 blocks per picture-category (BEGIN-smoking-stimuli, END-smoking-stimuli, BEGIN-control-stimuli, END-control-stimuli, erotic pictures, aversive pictures, pictures of every day activities) and with the constraint of not showing the same picture category twice in a row. Each block consisted of 15 pictures of only one picture category and each picture in a block was presented for 3 s. Thus, one block had a duration of 45 s. These four blocks of each picture category always contained the same pictures but in a different, randomly arranged order.

At the end of each block, subjective craving, valence, and arousal experienced during picture presentation were rated using a three button keypad (left, right, enter). For valence and arousal, a computerized version of the Self Assessment Manikin (SAM [51]) was applied. For craving, the scale was visualized with bars of different heights (amount of craving).

### Questionnaires

The Fagerström Test of Nicotine Dependence (FTND [52]) was used to assess the severity of addiction. Baseline craving was assessed with the Questionnaire on Smoking Urges (QSU-G [53]), smoking history with a self-constructed questionnaire. This smoking history questionnaire as well as two questionnaires developed in the framework of the transtheoretical model of behavior change (‘readiness to change questionnaire’ [54]; ‘decisional balance questionnaire’ [55]) were used to further validate the self-categorization of the subjects in consonant and dissonant smokers. The smoking history questionnaire contained the following questions relevant for the concept of consonant and dissonant smokers:

1. On average, how many cigarettes per day have you smoked over the last 12 months?
2. How long have you been smoking (in months)?
3. Have you ever tried to quit or to reduce smoking? (1 = yes, 2 = no).
4. How often have you tried to quit smoking?
5. Are you trying to quit or to reduce smoking at the moment? (1 = yes, 2 = no).
6. Do you think that you are addicted to cigarettes? (1 = yes, 2 = no).
7. Do you think about reasons why it would be better to quit smoking? (1 = yes, 2 = no).
8. Is smoking a pleasure for you? (1 = yes, 2 = no).
9. Would you stop smoking if you could do so easily? (1 = yes, 2 = no).
10. How satisfied are you with yourself being a smoker? (9-point likert scale ranging from 1 = very dissatisfied to 9 =  very satisfied).

The additional two questionnaires developed in the framework of the transtheoretical model of behavior change were the ‘readiness to change questionnaire’ [54], which comprises the subscales ‘precontemplation’ (i.e. the tendency of not thinking about quitting), ‘contemplation’ (i.e. the tendency of thinking about quitting), and ‘action’ (i.e. the tendency of actually trying to quit), and the ‘decisional balance questionnaire’ [55], which assesses the experienced advantages and disadvantages of smoking.

### Procedure

To begin with, the study was explained to the participants, possible contraindications were checked, and written consent was obtained. Thereafter, each participant had to smoke one cigarette. This was done to ensure that the subjects had an equal degree of satiation. Then, all questionnaires had to be filled in. Exhaled carbon monoxide (CO) was measured with a Micro 4 Smokerlyzer (Bedfont Scientific Ltd; http://www.bedfont.com/smokerlyzer). For familiarization with the rating procedure, training-trials with neutral pictures (IAPS [50]) took place outside and inside the scanner just before the experiment started. After the experiment, all subjects were debriefed and compensated financially (10 Euro/h) or with course credits.

### Image acquisition and analysis

#### Basic acquisition parameters

Brain images were acquired using a 1.5 T whole-body tomograph (Siemens Symphony) with a standard head coil. Anatomical measurements consisted of 160 T1-weighted sagittal images (MPRage, 1 mm slice thickness). A gradient echo field map sequence was used for assessing B0 distortions. For functional imaging, 780 volumes (3 dummy-scans) were registered using a T2*-weighted gradient echo-planar imaging sequence (EPI) with 25 transversal slices (parallel to AC-PC line) covering the whole brain (slice thickness  = 5 mm; 1 mm gap; descending; TR  =  2.5 s; TE  =  55 ms; flip angle  =  90°; FOV  =  192×192 mm; matrix  = 64×64).

#### Preprocessing

The statistical parametric mapping software package (SPM8, Wellcome Department of Cognitive Neurology, London, UK) implemented in Matlab (Mathworks, Natick, MA, Release 2007b) was used for preprocessing and statistical analyses. Origin coordinates were adjusted to the anterior commissure (AC). Realignment and unwarping (third-order B-spline), slice time correction, and normalization to the standard brain of the Montreal Neurological Institute were performed. Smoothing was executed with an isotropic three-dimensional Gaussian filter with a full width at half maximum of 9 mm.

#### First level analysis

For the first level analysis, all experimental conditions and ratings were modeled with boxcars convolved with the canonical hemodynamic response function in a General Linear Model. The six movement parameters of the rigid body transformation applied by the realignment procedure were introduced as covariates in the model. Serial correlations in the voxel-based time series were considered as a first-order autoregressive process and used for pre-whitening. A high-pass filter (time constant  = 360 s) was implemented using cosine functions in the design matrix. Previous research has shown that BEGIN- and END-smoking-stimuli differ at least in the strength of the elicited responses (strong responses elicited by BEGIN-smoking-stimuli, only weak responses elicited by END-smoking-stimuli). Further, it was suggested that END-smoking-stimuli might be a distinct stimulus class eliciting unique response patterns that might be opposite to responses elicited by BEGIN-smoking-stimuli [24], [32]–[35]. Given these differences, BEGIN-smoking-stimuli and END-smoking-stimuli were analyzed separately. For analyses of hemodynamic responses, contrasts between smoking-stimuli and their corresponding control stimuli were calculated for each subject. These contrasts were then used as dependent variables on the second level. As it was the case in our previous research [34], [35], activations were defined as positive differences between smoking and control stimuli; deactivations were defined as negative differences.

#### Second level analysis

The second level analyses comprised within group T-tests as well as between group T-tests regarding the described within subject difference contrasts (smoking *minus* control). Regions of interest anatomical masks were created using the Harvard-Oxford Cortical and Subcortical probabilistic atlases included in FSL (http://www.fmrib.ox.ac.uk/fsl/). Regions of interest were mesolimbic and mesocortical structures known to be part of a neuronal addiction network [5], [8], [9], [26]–[28]: nucleus accumbens (Nacc), orbitofrontal cortex (OFC), subcallosal cortex (SUBCC), medial frontal cortex (MFC), insula, amygdala, hippocampus, medial frontal gyrus (MFG, part of the dorsolateral prefrontal cortex) and anterior cingulate cortex (ACC). Only voxels, which belong to the respective region with ≥25% probability and additionally do not show a higher probability for belonging to another brain region, were included. Results are reported for a FWE corrected voxel level significance threshold of α = .05 [56].

### Analyses of subjective data

Subjective data were analyzed with SPSS 17.0 (http://www.spss.com).

With regard to stimulus-ratings, t-tests were used to compare stimuli or groups. With regard to group comparisons of stimulus ratings, we calculated within group differences between measures assessed in the smoking-stimulus condition *minus* the corresponding control stimulus condition and compared the groups for these differences. This was done to ensure that results are based only on the smoking content of the pictures and not on baseline differences between groups (e.g. baseline differences in craving).

Regarding questionnaire data, t-tests were used for group comparisons of the scales of the readiness to change questionnaire [54], the decisional balance questionnaire [55], as well as items 1, 2, and 4 of the smoking history questionnaire. Chi-square tests were used for group comparisons of items 3, 5, 6, 7, 8, and 9 of the smoking history questionnaire.

## Results

One consonant smoker and two dissonant smokers had to be excluded due to technical problems during normalization of the fMRI images. Accordingly, all analyses are based on 15 consonant and 14 dissonant smokers.

### Subject characteristics

Subject characteristics are summarized in Table 1 and Table 2.

**Table 1:** Mean (SD) for subject demographics, self-rated attitudes and the scales of the readiness to change questionnaire and the decisional balance questionnaire (attitude scales).

	ENTIRE SAMPLE (15m, 14f)	CONSONANT SMOKERS (7m, 8f)	DISSONANT SMOKERS (8m, 6f)	*t*	*p*
***Subject demographics*** **:**					
**Age (years)**	24.55 (3.48)	24.60 (3.87)	24.50 (3.16)	0.08	.940
**Years smoked**	8.63 (3.56)	9.33 (3.64)	7.89 (3.43)	1.09	.283
**Cigarettes/day**	17.93 (3.35)	16.97 (2.43)	18.96 (3.94)	−1.66	.109
**FTND**	4.07 (1.96)	4.20 (1.61)	3.93 (2.34)	0.37	.717
**QSU**	3.21 (1.13)	3.50 (1.33)	2.90 (0.81)	1.44	.160
**CO**	27.48 (7.35)	26.27 (6.03)	28.79 (8.59)	−0.92	.366
***Attitude Scales:***					
**CON/DIS value**	5.07 (2.75)	2.53 (0.52)	7.79 (0.81)	−21.12	<.001
**Satisfaction**	4.97 (2.56)	7.20 (1.15)	2.57 (0.85)	12.27	<.001
**Number of quit attempts**	2.50 (3.81)	2.80 (5.13)	2.00 (1.36)	0.57	.580
Advantages *	29.35 (7.50)	30.53 (7.87)	28.0 (7.14)	0.88	.387
Disadvantages *	29.93 (8.51)	24.87 (8.01)	35.3 (5.05)	−4.18	<.001
**Precontemplation ^†^**	−0.55 (2.32)	0.60 (2.13)	−1.79 (1.89)	3.18	.004
**Contemplation ^†^**	2.00 (4.48)	−0.20 (4.59)	4.36 (3.00)	−3.19	.004
**Action ^†^**	−4.83 (3.72)	−7.00 (1.89)	−2.50 (3.84)	−3.96	.001

***Remarks.*** t  =  t-value for the comparison of consonant and dissonant smokers. p  =  p value for the comparison of consonant and dissonant smokers. FTND  =  Fagerström test of nicotine dependence, QSU  =  Questionnaire on smoking urges, CO  =  CO value prior to the experiment, CON/DIS value  =  self characterization on a 9-point-likert-scale, satisfaction  =  response to the question ‘how satisfied are you with yourself being a smoker?’ from the smoking history questionnaire, number of quit attempts  =  response to the question ‘How often have you tried to quit smoking?’ from the smoking history questionnaire, *  =  scales of the decisional balance questionnaire (advantages  =  perceived advantages of smoking, disadvantages  =  perceived disadvantages of smoking), †  =  scales of the readiness to change questionnaire (precontemplation  =  tendency of not thinking about quitting, contemplation  =  tendency of thinking about quitting, action  =  tendency of actually trying to quit).

**Table 2:** Subject demographics for the items of the smoking history questionnaire that were analyzed with chi square tests.

	Consonant Smokers		Dissonant Smokers		χ^2^	p
	Yes	No	Yes	No		
**Have you ever tried toquit or to reduce smoking?**	11	4	14	0	4.33	.037
**Are you trying to quit or to reduce smoking at the moment?**	1	14	10	4	12.90	<.001
**Do you think that you are addicted?**	15	0	14	0	-	-
**Do you think about reasonsto quit smoking?**	5	10	14	0	14.25	<.001
**Is smoking a pleasurefor you?**	14	1	10	4	2.44	.119
**Would you stop if you coulddo so easily?**	1	14	14	0	25.26	<.001

Digits show the number of subjects who chose a certain response.

The following data can be seen in Table 1. The subjects’ self-categorization revealed consonant smokers having a mean dissonance value of 2.53 (i.e. being quite consonant) and dissonant smokers having a mean dissonance value of 7.79 (i.e. being quite dissonant). As expected, both groups differed significantly in this dissonance value (*t*(27) =  −21.12, *p*<.001). Both groups did not differ in age, degree of addiction (FTND), actual craving (QSU), or CO-values. In response to the question ‘How satisfied are you with yourself being a smoker?’ consonant smokers had a mean value of 7.20 (i.e. being satisfied) whereas dissonant smokers had a mean value of 2.57 (i.e. being dissatisfied). Here, both groups differed significantly (*t*(27) = 12.27, *p*<.001). Concerning the two questionnaires developed in the framework of the transtheoretical model of behavior change (‘readiness to change questionnaire’ [54]; ‘decisional balance questionnaire’ [55]), the decisional balance questionnaire revealed dissonant smokers perceiving more disadvantages of smoking than consonant smokers (*t*(27) = 4.18, *p*<.001). Also, dissonant smokers perceived more disadvantages than advantages (*t*(13) = −3.82, *p* = .002), whereas consonant smokers perceived more advantages than disadvantages of smoking (*t*(14) = 2.75, *p* = .016). However, no group-differences occurred for the perceived advantages, which is surprising given that consonant smokers are assumed to be content with their smoking behavior. The readiness to change questionnaire revealed that dissonant smokers had higher values on the contemplation scale (*t*(24.30) = 3.19, *p* = .004) and the action scale (*t*(18.66) = 3.96, *p* = .001) than consonant smokers. Consonant smokers showed higher values on the precontemplation scale than dissonant smokers (*t*(27) = 3.18, *p* = .004).

Table 2 shows the items of the smoking history questionnaire that were analyzed with chi square tests. Dissonant smokers were more likely to state that they 1) would stop smoking if they could do so easily (*χ^2^* = 25.26, *p*<.001), 2) think about reasons to quit (*χ^2^* = 14.15, *p*<.001), 3) tried to quit or to reduce the amount of smoking in the past (*χ^2^* = 4.33, *p* = .037), and 4) are trying to quit or to reduce the amount of smoking at the moment (*χ^2^* = 12.90, *p*<.001).

Taken together, the questionnaire-data confirmed the subjects' self-attribution of different attitudes towards their smoking behavior. However, no group differences occurred for 1) amount of smoking, 2) number of attempts to quit, 3) self-attribution of addiction, or 4) perception of smoking as pleasurable.

### Ratings

All ratings are summarized in Table 3 and Table 4.

**Table 3:** Mean (SD) for stimulus ratings for BEGIN-smoking and BEGIN-control-stimuli.

RATING SCALE	GROUP	BEGIN-SMOKING- STIMULI	BEGIN-CONTROL- STIMULI	t	p
**CRAVING**					
	**ENTIRE SAMPLE**	5.86 (1.75)	4.46 (1.91)	5.69	<.001
	**CONSONANT SMOKERS**	6.29 (1.60)	5.00 (2.00)	3.95	.001
	**DISSONANT SMOKERS**	5.39 (1.84)	3.88 (1.68)	4.00	.002
**VALENCE**					
	**ENTIRE SAMPLE**	5.06 (1.22)	5.39 (1.27)	−1.56	.129
	**CONSONANT SMOKERS**	5.40 (1.18)	5.66 (1.27)	−0.85	.410
	**DISSONANT SMOKERS**	4.69 (1.78)	5.11 (1.26)	−1.34	.203
**AROUSAL**					
	**ENTIRE SAMPLE**	4.16 (1.35)	3.51 (1.42)	3.31	.003
	**CONSONANT SMOKERS**	4.13 (1.21)	3.36 (1.36)	2.48	.026
	**DISSONANT SMOKERS**	4.20 (1.53)	3.69 (1.52)	2.18	.049

**Table 4:** Mean (SD) for stimulus ratings for END-smoking and END-control-stimuli.

RATING SCALE	GROUP	END-SMOKING- STIMULI	END-CONTROL- STIMULI	t	p
**CRAVING**					
	**ENTIRE SAMPLE**	4.95 (1.62)	4.55 (1.71)	2.33	.027
	**CONSONANT SMOKERS**	5.29 (1.61)	5.03 (1.79)	1.62	.127
	**DISSONANT SMOKERS**	4.58 (1.60)	4.04 (1.53)	1.76	.103
**VALENCE**					
	**ENTIRE SAMPLE**	4.98 (1.08)	5.50 (1.14)	−2.57	.016
	**CONSONANT SMOKERS**	5.17 (1.23)	5.73 (1.15)	−2.10	.055
	**DISSONANT SMOKERS**	4.77 (0.89)	5.26 (1.12)	−1.52	.153
**AROUSAL**					
	**ENTIRE SAMPLE**	4.04 (1.40)	3.37 (1.40)	3.93	.001
	**CONSONANT SMOKERS**	3.83 (1.30)	3.25 (1.32)	2.28	.039
	**DISSONANT SMOKERS**	4.25 (1.52)	3.50 (1.52)	3.33	.005

#### BEGIN-smoking-stimuli (Table 3)

No significant group differences occurred regarding the within subject difference scores (BEGIN-smoking-stimuli *minus* BEGIN-control-stimuli). In the entire sample, BEGIN-smoking-stimuli compared to BEGIN-control-stimuli led to more craving (*t*(28) = 5.69, *p*<.001) and were rated as more arousing (*t*(28) = 3.31, *p* = .003). Concerning valence ratings, BEGIN-stimuli and control stimuli did not differ.

#### END-smoking-stimuli (Table 4)

No significant group differences occurred regarding the within subject difference scores (END-smoking-stimuli *minus* END-control-stimuli). In the entire sample, END-smoking-stimuli led to more craving than END-control stimuli (*t*(28) = 2.33, *p* = .027). Further, END-smoking-stimuli had lower valence ratings than END-control-stimuli (*t*(28) = -2.57, *p* = .016). Regarding the arousal ratings, the entire sample showed higher arousal in response to END-smoking-stimuli than in response to END-control-stimuli (*t*(28) = 3.93, *p* = .001).

### Brain Data

All brain data are summarized in table 5 and table 6.

**Table 5:** Significant activations and deactivations for BEGIN-smoking-stimuli and significant differences between consonant and dissonant smokers in their responses to BEGIN-smoking-stimuli.

Contrast	Structures	Side	x	y	z	t	*p*_corr_
**DISSONANT SMOKERS**							
**ACTIVATIONS**							
	Amygdala	l	−15	−10	−17	4.14	.028
	Amygdala	r	27	2	−20	4.42	.020
	Hippocampus	l	−18	−16	−17	4.81	.025
	Insula	l	−30	−25	13	4.81	.039
**DEACTIVATIONS**							
	no significant results						
**DISSONANT SMOKERS vs. CONSONANT SMOKERS**							
**DISSONANT > CONSONANT**							
	Amygdala	l	−18	−4	−20	3.35	.037
	Amygdala	r	27	2	−20	4.97	.001
	Hippocampus	l	−24	−40	−5	3.66	.043
	Insula	l	−30	−25	16	4.57	.010
	Insula	r	33	5	13	4.84	.005
**CONSONANT > DISSONANT**							
	no significant results						

***Remarks.*** No significant results occurred for the entire sample or for consonant smokers.

#### BEGIN-smoking-stimuli (Table 5)

Significant activations (smoking > control) elicited by BEGIN-smoking-stimuli occurred neither in the entire sample nor in consonant smokers. Dissonant smokers, however, showed activations in left and right amygdala, left hippocampus, and left insula. Further, significant group differences occurred: Regions of interest analyses revealed dissonant smokers having stronger activations (smoking > control) than consonant smokers in left and right amygdala, left hippocampus, as well as left and right insula.

#### END-smoking-stimuli (Table 6)

In the entire sample, END-smoking-stimuli led to a significant activation (smoking > control) in the ACC. In dissonant smokers, a significant deactivation (smoking < control) was found in the left OFC. The group comparison revealed that the smoking minus control contrast was significantly smaller in dissonant smoker than in consonant smokers in the SUBCC. An analogous trend was found in the left OFC.

## Discussion

The present study investigated the influence of different attitudes towards ones own smoking behavior on subjective and neural responses to smoking associated stimuli. Consonant and dissonant smokers underwent an fMRI protocol. They were presented smoking stimuli from different stages of the intake ritual (BEGIN/END) as well as neutral stimuli (additionally, general emotional stimuli were presented that are not part of the reported analyses; see methods for details). As a major result, we found stronger responses in the “addiction network” in dissonant smokers than in consonant smokers.

First of all, the questionnaire data confirmed the conceptual distinctiveness of consonant and dissonant smokers. In contrast to consonant smokers, dissonant smokers were more likely to report that they would stop smoking if it could be done easily. Furthermore, dissonant smokers were less content with their smoking behavior, perceived more disadvantages, and were more likely to think about reasons for stopping as well as more likely to try to reduce the amount of smoking.

These findings are in accordance with Eiser, Sutton & Wober [10]. Contrary to these authors, however, no group differences were found for the severity of addiction or the self-attribution of addiction. Eiser et al. [10] did not investigate the actual severity of addiction; but they found that dissonant smokers were more likely than consonant smokers to consider themselves addicted. The authors interpret their finding as a form of dissonance reduction, i.e. dissonant smokers may justify their behavior with the belief that they are addicted. It has to be considered, however, that knowledge of the addiction potential of cigarette smoking has grown since the study by Eiser and colleagues was published in the late seventies [10]. Thus, today it may be difficult for all smokers to deny being addicted. Consequently, the consonant smokers of the present study might better fit the criteria for “happy” addicts; a classification that was introduced by Skog [11]. The author describes addicts on a consonance-dissonance dimension. On this dimension, consonant smokers as described by Eiser and colleagues [10] would be located on the absolute consonant side and would be called naïve addicts, because they deny being addicted. Smokers, who acknowledge being addicted but have a positive attitude towards their smoking habit, are called “happy” addicts. In addition, in the present study, the amount of smoking was very similar in the two subgroups. It seems therefore rather unlikely that the reported stimulus ratings and brain data are influenced by factors other than smoking attitude.

Concerning brain data, it was surprising that the BEGIN-smoking-stimuli did neither elicit mesocorticolimbic brain activity in the entire sample nor in consonant smokers, as found in our previous study [34]. Differences in study design might account for this (e.g. block-design with a rather long block-length). Even more impressive, dissonant smokers revealed significant activations in amygdala, hippocampus, and insula. These activations were, in fact, significantly increased compared to consonant smokers.

All three structures are crucially involved in motivational and emotional processing. In the context of addiction research, they are discussed as being part of a neuronal addiction network involved in cue detection, reactivation of drug memories, elicitation of appetitive psychophysiological responses to cues, and in the generation of a conscious experience of these responses [8], [9], [26]–[28].

**Table 6:** Significant activations and deactivations for END-smoking-stimuli and significant differences between consonant and dissonant smokers in their responses to END-smoking-stimuli.

Contrast	Structures	Side	x	y	z	t	*p*_corr_
**ENTIRE SAMPLE**							
**ACTIVATIONS**							
	ACC	l	−3	8	43	4.10	.042
**DEACTIVATIONS**							
	no significant results						
**DISSONANT SMOKERS**							
**ACTIVATIONS**							
	no significant results						
**DEACTIVATIONS**							
	OFC	l	−18	17	−20	5.14	.029
**DISSONANT SMOKERS vs. CONSONANT SMOKERS**							
**DISSONANT > CONSONANT**							
	no significant results						
**CONSONANT > DISSONANT**							
	SUBCC	r	3	14	−20	4.01	.024
	OFC	l	−39	32	−5	3.66	.072

***Remarks.*** No significant results occurred for consonant smokers.

The amygdala is known to be involved in processing the reward value of various kinds of learned and unlearned stimuli [57]–[60]. It has been proposed that in addiction the amygdala has an important role in the detection of drug cues and shows an abnormal activity in response to these cues (e.g. [28], [61]). According to Bechara [5], it is an important part of the neuronal system underlying the impulsive processing of drug cues in addicts. Further, it has been suggested that the amygdala is particularly important in cue-induced relapse [62]. Interestingly, a current meta-analysis regarding cue reactivity in a wide array of addictions revealed the amygdala-hippocampus system to be more reliably activated in addicts with high motivation to quit than in addicts with no motivation to quit [29]. This has been interpreted in terms of a reactivation of drug memories [29].

According to the somatic marker hypothesis [63], [64], affective responses to salient stimuli are evoked by amygdala projections to structures changing the internal milieu of the body, such as visceral motor structures and certain brainstem nuclei, as well as through behaviour related structures such as the ventral striatum. The internal changes elicited by the amygdala have further influences on neural processing and motivated behavior. One structure of critical importance in the further processing of these amygdala induced internal changes is the insula. The insula has important functions in interoception, i.e. the neural mapping of internal bodily states [65]–[67]. Furthermore, the right anterior insula is thought to be the place where conscious awareness of internal processes arises, which in turn is proposed to be an important part of emotion [63]–[67]. The structure's importance for addiction has been illustrated by a human study demonstrating that subjects with lesions to the insula (compared with subjects suffering from other brain damages) were able to quit smoking immediately [68] as well as an animal study showing that inactivating the insula leads to a disruption of drug conditioned place preference in rats [69]. Based upon this body of literature, Naqvi and Bechara [8], [9] propose a model of insula function in addiction (see also [5]). According to the authors, the insula processes interoceptive states that are produced by drugs and integrates these into conscious feelings and into decision-making processes. Whereas many of the drug induced bodily processes are aversive at first, dopaminergic modulation of insula, amygdala, and ventromedial prefrontal cortex is suggested to turn them into very strong internal incentive stimuli later on. Similar to Damasio [63], [64], Naqvi and Bechara [8], [9] propose that these interoceptive processes can also be triggered (without ingestion of a drug) by a stimulus associated with drug consumption. As noted above, the amygdala is critical for this. These processes are encoded by the insula and integrated into motivational processes (like the emergence of craving [70]) that support addiction and bias decision making, which might lead to relapses.

With this in mind, our results point towards an enhanced incentive processing of BEGIN-smoking-stimuli in dissonant smokers. The neural impulsive system of this group of smokers might be hyper-responsive to drug-cues, which could in turn lead to exaggerated incentive motivational responses encoded by the insula. One could further speculate that this might then weaken the counteracting reflective system, diminish the influence of reflective reasoning about the danger of smoking, and make it difficult for dissonant smokers to quit smoking. Given this, the hyper-responsivity could explain why consonant and dissonant smokers did not differ in ratings, amount of smoking, number of attempts to quit, and perception of smoking as pleasurable. At the moment, this has to stay speculation of course. However, similar results showing group differences in physiological but not subjective measures have been reported previously [23], [49].

Regarding the END-smoking-stimuli results, similar interpretations seem conceivable. When looking only at dissonant smokers, we found a deactivation in the OFC. Further, SUBCC activity in response to END-smoking-stimuli (minus END-control-stimuli) was significantly lower in dissonant smokers than in consonant smokers. Keeping in mind that such deactivations could point to a unique reactivity of END-smoking-stimuli, which might have a specific function in the guidance of behavior [24], this result seems to further emphasize that dissonant smokers process drug associated stimuli more impulsively. Contrary to our earlier study [34], however, we did not find any deactivations in the ventral striatum or the ACC. Surprisingly, the ACC was activated by END-smoking-stimuli when analysing the entire sample. Thus, results of our earlier study [34] could not be confirmed here. It is possible that the different study designs account for the diverging results.

### Limitations

Two potential limitations were noted by the reviewers and need to be addressed.

First, the current analysis is part of a larger study in which also aversive and erotic pictures were presented, in addition to the smoking stimuli analyzed here. Theoretically, it could be possible that these stimuli influenced the responses to the smoking stimuli. However, to us this seems to be very unlikely because the study was designed in a way that should prevent such influences (i.e. having no overlap between regressors, randomization of picture categories). Nevertheless it might be sensible to conduct further research on the influence of attitudes on the processing of smoking stimuli without presenting such additional stimuli.

Second, the low number of subjects per group need to be considered. This might have reduced the power of our experiment and we might have missed some additional effects. Nevertheless, the effects that are significant despite insufficient power must have a considerable size to become significant and should therefore not only be seen as reliable, but also as quantitatively stronger than the same results with a larger sample size.

### Conclusions

In sum, our study was able to demonstrate that the attitude towards ones cigarette consumption behavior can influence the neural processing of smoking stimuli. The present results suggest that the incentive value of drug-associated stimuli stimulates dissonant users more than consonant users. Rather speculatively, a possibly enhanced reflective processing of the negative consequences of smoking might counteract these impulsive processes. As a result, the observable behavior of dissonant smokers might be not distinguishable from that of consonant smokers.

## References

[1] Koob GF, Le Moal M (1997) Drug Abuse: Hedonic Homeostatic Dysregulation. Science 3: 52–58. 10.1126/science.278.5335.52 9311926

[2] Hyman SE, Malenka RC (2001) Addiction and the brain: the neurobiology of compulsion and its persistence. Nature Reviews: Neuroscience 2: 695–703. 11584307 10.1038/35094560

[3] Kalivas PW, Volkow ND (2005) The neural basis of addiction: a pathology of motivation and choice. Am J Psychiatry 162: 1403–1413. 16055761 10.1176/appi.ajp.162.8.1403

[4] Robinson TE, Berridge KC (1993) The neural basis of drug craving: an incentive-sensitization theory of addiction. Brain Research and Brain Research Reviews 18: 247–291. 8401595 10.1016/0165-0173(93)90013-p

[5] Bechara A (2005) Decision making, impulse control and loss of willpower to resist drugs: a neurocognitive perspective. Nature Neuroscience 8: 1458–1463. 16251988 10.1038/nn1584

[6] Strack F, Deutsch R (2004) Reflective and Impulsive Determinants of Social Behavior. Personality and Social Psychology Review 8: 220–247. 15454347 10.1207/s15327957pspr0803_1

[7] Deutsch R, Strack F (2006) Reflective and Impulsive Determinants of Addictive Behavior. In: Wiers RW, Stacy AW (Eds). Handbook of implicit cognition and addiction. (45 57). Thousand Oaks, CA, US: Sage Publications.

[8] Naqvi NH, Bechara A (2009) The hidden island of addiction: the insula. Trends Neurosci 32: 56–67. 18986715 10.1016/j.tins.2008.09.009 PMC3698860

[9] Naqvi NH, Bechara A (2010) The insula and drug addiction: An interoceptive view of pleasure, urges, and decision-making. Brain Structure & Function 214: 435–450. 20512364 10.1007/s00429-010-0268-7 PMC3698865

[10] Eiser JR, Sutton SR, Wober M (1978) “Consonant” and “dissonant” smokers and the self-attribution of addiction. Addictive Behaviors 3: 99–106. 717098 10.1016/0306-4603(78)90032-1

[11] Skog O (2003) Addiction: Definitions and mechanisms. In Vuchinich RE, Heather N (Eds.). Choice, behavioral economics and addiction. (157–175). New York: The Guilford Press.

[12] Prochaska JO, DiClemente CC (1983) Stages and processes of self-change of smoking: Toward an integrative model of change. Journal of Consulting and Clinical Psychology 51: 390–395. 6863699 10.1037//0022-006x.51.3.390

[13] McCoy SB, Gibbons FX, Reis TJ, Gerrard M, Luus CAE, et al (1992) Perceptions of smoking risk as a function of smoking status. Journal of Behavioral Medicine 15: 469–488. 1447758 10.1007/BF00844942

[14] Prochaska JO, Velicer WF (1997) The transtheoretical model fo health behavior change. American Journal of Health Promotion 12: 38–48. 10170434 10.4278/0890-1171-12.1.38

[15] McKee SA, ÓMalley SS, Salovey P, Krishnan-Sarin S, Mazure CM (2005) Perceived risks and benefits of smoking cessation: gender-specific predictors of motivation and treatment outcome. Addictive Behaviors 30: 423–435. 15718060 10.1016/j.addbeh.2004.05.027

[16] Chouinard M-C, Robichaud-Ekstrand S (2007) Predictive value of the transtheoretical model to smoking cessation in hospitalized patients with cardiovascular disease. European Journal of Cardiovascular Prevention & Rehabilitation 14: 51–58. 17301627 10.1097/HJR.0b013e328014027b

[17] Stewart J, De Wit H, Eikelboom R (1984) Role of conditioned and unconditioned drug effects in the self-administration of opiates and stimulants. Psychological Review 91: 251–268. 6571424

[18] Robinson TE, Berridge KC (2003) Addiction. Annual Reviews Psychology 54: 25–53. 10.1146/annurev.psych.54.101601.145237 12185211

[19] Drummond D (2001) Theories of drug craving, ancient and modern. Addiction 96: 33–46. 11177518 10.1046/j.1360-0443.2001.961333.x

[20] Hyman SE (2005) Addiction: a disease of learning and memory. American Journal of Psychiatry 162: 1414–1422. 16055762 10.1176/appi.ajp.162.8.1414

[21] Carter BL, Tiffany ST (1999) Meta-analysis of cue-reactivity in addiction research. Addiction 94: 327–340. 10605857

[22] Geier A, Pauli P, Mucha RF (2000) Appetitive nature of drug cues confirmed with physiological measures in a model using pictures of smoking. Psychopharmacology 150: 283–291. 10923756 10.1007/s002130000404

[23] Dempsey JP, Cohen LM, Hobson VL, Randall PK (2007) Appetitive nature of drug cues re-confirmed with physiological measures and the potential role of stage of change. Psychopharmacology 194: 253–260. 17588224 10.1007/s00213-007-0839-3

[24] Mucha RF, Pauli P, Weber M, Winkler M (2008) Smoking stimuli from the terminal phase of cigarette consumption may not be cues for smoking in healthy smokers. Psychopharmacology 201: 81–95. 18704373 10.1007/s00213-008-1249-x

[25] Winkler MH, Weyers P, Mucha RF, Stippekohl B, Stark R, et al (2011) Conditioned cues for smoking elicit preparatory responses in healthy smokers. Psychopharmacology 213: 781–789. 20953588 10.1007/s00213-010-2033-2 PMC3033511

[26] Volkow ND, Fowler JS, Wang GJ (2003) The addicted human brain: insights from imaging studies. J Clin Investig 111: 1444–1451. 12750391 10.1172/JCI18533 PMC155054

[27] Volkow ND, Fowler JS, Wang GJ (2004) The addicted human brain viewed in the light of imaging studies: brain circuits and treatment strategies. Neuropharmacology 47(S1): 3–13. 15464121 10.1016/j.neuropharm.2004.07.019

[28] Everitt BJ, Robbins TW (2005) Neural systems of reinforcement for drug addiction: from actions to habits to compulsion. Nature Neuroscience 8: 1481–1489. 16251991 10.1038/nn1579

[29] Chase HW, Eickhoff SB, Laird AR, Hogarth L (2011) The neural basis of drug stimulus processing and craving: an activation likelihood estimation meta-analysis. Biological Psychiatry 70: 785–793. 21757184 10.1016/j.biopsych.2011.05.025 PMC4827617

[30] Kühn S, Gallinat J (2011) Common biology of craving across legal and illegal drugs – a quantitative meta-analysis of cue-reactivity brain response. Behavioral Neuroscience 33: 1318–1326. 10.1111/j.1460-9568.2010.07590.x 21261758

[31] Engelmann JM, Versace F, Robinson JD, Minnix JA, Lam CY, et al (2012) Neural substrates of smoking cue reactivity: a meta-analysis of fMRI studies. NeuroImage 60: 252–262. 22206965 10.1016/j.neuroimage.2011.12.024 PMC3288122

[32] Mucha RF, Geier A, Pauli P (1999) Modulation of craving by cues having differential overlap with pharmacological effect: Evidence for cue approach in smokers and social drinkers. Psychopharmacology 147: 306–113. 10639690 10.1007/s002130051172

[33] Mucha RF, Pauli P, Weyers P (2006) Psychophysiology and implicit cognition in drug use: significance and measurement of motivation for drug use with emphasis on startle tests. In: Wiers RW, Stacey AW, eds. Handbook of Implicit Cognition and Addiction,. 201–214. Thousand Oaks, CA: Sage.

[34] Stippekohl B, Winkler M, Mucha RF, Pauli P, Walter B, et al (2010) Neural responses to BEGIN- and END-stimuli of the smoking ritual in nonsmokers, nondeprived smokers, and deprived smokers. Neuropsychopharmacology 35: 1209–1255. 20090671 10.1038/npp.2009.227 PMC3055400

[35] Stippekohl B, Walter B, Winkler M, Mucha RF, Pauli P, et al.. (2012) An early attentional bias to BEGIN-stimuli of the smoking ritual is accompanied with mesocorticolimbic deactivations in smokers. Psychopharmacology. In press. 10.1007/s00213-012-2670-8 22476609

[36] Mucha RF, Geier A, Stuhlinger M, Mundle G (2000) Appetitive effects of drug cues modeled by pictures of the intake ritual: generally of cue-modulated startle examined with inpatient alcoholics. Psychopharmacology 151: 428–432. 11026750 10.1007/s002130000508

[37] Nees F, Diener C, Smolka MN, Flor H (2011) The role of context in the processing of alcohol-relevant cues. Addiction Biology 17: 441–451. 21790904 10.1111/j.1369-1600.2011.00347.x

[38] Benowitz NL (1990) Clinical pharmacology of inhaled drugs of abuse: implications in understanding nicotine dependence. NIDA Research Monograph 99: 12–29. 2267009

[39] Rose JE, Behm FM, Westman EC, Coleman RE (1999) Arterial nicotine kinetics during cigarette smoking and intravenous nicotine administration: implications for addiction. Drug and Alcohol Dependence 56: 99–107. 10482401 10.1016/s0376-8716(99)00025-3

[40] Jarvik ME, Madsen DC, Olmstead RE, Iwamoto-Schaap PN, Elins JL, et al (2000) Nicotine Blood Levels and Subjective Craving for Cigarettes. Pharmacology Biochemistry and Behavior 66: 553–558. 10.1016/s0091-3057(00)00261-6 10899369

[41] Carter BL, Tiffany ST (2001) The cue-availability paradigm: the effects of cigarette availability on cue reactivity in smokers. Experimental and Clinical Psychopharmacology 9: 183–190. 11518094 10.1037//1064-1297.9.2.183

[42] Wertz JM, Sayette MA (2001) Effects of smoking opportunity on attentional bias in smokers. Psychology of Addictive Behaviors 15: 268–271. 11563808 PMC2632777

[43] Wertz JM, Sayette MA (2001b) A review of the effects of perceived drug use opportunity on self-reported urge. Experimental and Clinical Psychopharmacology 9: 3–13. 11519632 10.1037/1064-1297.9.1.3 PMC2632967

[44] Wilson SJ, Sayette MA, Fiez JA (2004) Prefrontal responses to drug cues: a neurocognitive analysis. Nature Neuroscience 7: 211–214. 15001989 10.1038/nn1200 PMC2637355

[45] McBride D, Barret SP, Kelly JT, Aw A, Dagher A (2006) Effects of expectancy and abstinence on the neural response to smoking cues in cigarette smokers: an fMRI study. Neuropsychopharmacology 31: 2728–2738. 16598192 10.1038/sj.npp.1301075

[46] Bailey SR, Goedeker KC, Tiffany ST (2010) The impact of cigarette deprivation and cigarette availability on cue reactivity in smokers. Addiction 105: 364–372. 19922514 10.1111/j.1360-0443.2009.02760.x PMC2807894

[47] Lubman DJ, Yücel M, Pantelis C (2004) Addiction, a condition of compulsive behaviour? Neuroimaging and neuropsychological evidence of inhibitory dysregulation. Addiction 99: 1491–1502. 15585037 10.1111/j.1360-0443.2004.00808.x

[48] Muñoz MS, Idrissi S, Sánchez-Barrera MB, Fernández MC, Vila J (2011) Motivation to quit smoking and startle modulation in female smokers: context specificity of smoking cue reactivity. Psychopharmacology 218: 1–8. 21594561 10.1007/s00213-011-2334-0

[49] McDermut W, Haaga DAF (1998) Effect of stage of change on cue reactivity in continuing smokers. Experimental and Clinical Psychopharmacology 6: 316–324. 9725115 10.1037//1064-1297.6.3.316

[50] Lang PJ, Bradley MM, Cuthbert B (1995) International Affective Picture System. Center for Research in Psychophysiology, University of Florida: Gainsville, Florida.

[51] Bradley MM, Lang PJ (1994) Measuring emotion: the self-assessment manikin and the semantic differential. J Behav Ther Exp Psychiatry 25: 49–59. 7962581 10.1016/0005-7916(94)90063-9

[52] Schumann A, Rumpf HJ, Meyer C, Hapke U, John U (2003) Deutsche Version des Fagerström-Test for Nicotine Dependence (FTND-G) und des Heaviness of Smoking Index (HSI-G). In: Glöckner-Rist A, Rist F, & Küfner H, editors. Elektronisches Handbuch zu Erhebungsinstrumenten im Suchtbereich (EHES). Version 3.00. Mannheim: Zentrum für Umfragen, Methoden und Analysen.

[53] Müller V, Mucha RF, Ackermann K, Pauli P (2001) Die Erfassung des Cravings bei Rauchern mit einer deutschen Version des „Questionnaire on Smoking Urges”. Z Klin Psychol Psychother 30: 164–171.

[54] Hannöver W, Thyrian JR, Rumpf HJ, Meyer C, Hapke U, et al.. (2003) Der Fragebogen zur Änderungsbereitschaft bei Rauchern (FÄR). In A. Glöckner-Rist, F. Rist, & H. Küfner (Hrsg.), Elektronisches Handbuch zu Erhebungsinstrumenten im Suchtbereich (EHES). Version 3.00. Mannheim: Zentrum für Umfragen, Methoden und Analysen.

[55] Schumann A, Rumpf HJ, Meyer C, Hapke U, John U (2003) Deutsche Version des Fragebogens zur Decisional Balance für Raucher (DBR-G). In A. Glöckner-Rist, F. Rist, & H. Küfner (Hrsg.), Elektronisches Handbuch zu Erhebungsinstrumenten im Suchtbereich (EHES). Version 3.00. Mannheim: Zentrum für Umfragen, Methoden und Analysen.

[56] Worsley KJ (2001) Statistical analysis of activation images. In Jezzard P, Matthews PM, Smith SM (eds). Functional MRI: An Introduction to methods. Oxford University Press: Oxford. 251–270.

[57] Baxter MG, Murray EA (2002) The amygdala and reward. Nature Reviews: Neuroscience 3: 563–573. 12094212 10.1038/nrn875

[58] Dalgleish T (2004) The emotional brain. Nature Reviews: Neuroscience 5: 582–589. 10.1038/nrn1432 15208700

[59] Martin-Soelch C, Linthicum J, Ernst M (2007) Appetitive conditioning: Neural bases and implications for psychopathology. Neuroscience & Biobehavioral Reviews 31: 426–440. 17210179 10.1016/j.neubiorev.2006.11.002 PMC2693132

[60] Pauli WM, Hazy TE, ÓReilly RC (2011) Expectancy, Ambiguity, and Behavioral Flexibility: Separable and Complementary Roles of the Orbital Frontal Cortex and Amygdala in Processing Reward Expectancies. Journal of Cognitive Neuroscience 24: 351–366. 22004047 10.1162/jocn_a_00155

[61] Everitt BJ, Parkinson JA, Olmstead MC, Arroyo M, Robledo P, et al (1999) Associative processes in addiction and reward: the role of amygdala and ventral striatal subsystems Annals of the New York Acadamey of Sciences. 877: 412–438. 10.1111/j.1749-6632.1999.tb09280.x 10415662

[62] See RE, Fuchs RA, Ledford CC, McLaughlin J (2003) Drug addiction, relapse, and the amygdala. Ann NY Acad Sci 985: 294–307. 12724166 10.1111/j.1749-6632.2003.tb07089.x

[63] Damasio AR (1994) Descartes' Error: emotion, reason and the human brain. Putnam and Sons, New York.

[64] Damasio AR (2000) The Feeling of What Happens: Body and Emotion in the Making of Consciousness. Harcourt, New York.

[65] Craig AD (2002) How do you feel? Interoception: the sense of the physiological condition of the body. Nature Reviews Neuroscience 3: 655–666. 12154366 10.1038/nrn894

[66] Craig AD (2003) Interoception: the sense of the physiological condition of the body. Current Opinion in Neurobiology 13: 500–505. 12965300 10.1016/s0959-4388(03)00090-4

[67] Craig AD (2009) How do you feel – now? The anterior insula in human awareness. Nature Reviews: Neuroscience 10: 59–70. 19096369 10.1038/nrn2555

[68] Naqvi NH, Rudrauf D, Damasio H, Bechara A (2007) Damage to the insula disrupts addiction to cigarette smoking. Science 315: 531–534. 17255515 10.1126/science.1135926 PMC3698854

[69] Contreras M, Ceric F, Torrealba F (2007) Inactivation of the interoceptive insula disrupts drug craving and malaise induced by lithium. Science 318: 655–658. 17962567 10.1126/science.1145590

[70] Garavan H (2010) Insula and drug cravings. Brain structure and function 214: 593–601. 20512373 10.1007/s00429-010-0259-8

